# Progress in Surgery

**Published:** 1897-07-03

**Authors:** 


### Progress in Surgery.

### SURGERY OF THE RECTUM.

**Operations for Carcinoma.**—In his Lettsomian lectures in 1896, upon Operations for Cancer, Mr. Watson Cheyne spoke in favour of the palliative operation of colotomy, and deprecated the extensive procedures which Continental surgeons advocate for extirpation of the diseased area in carcinoma of the rectum. But there are signs that this view of the matter will, for a considerable proportion of cases, cease to be a representative view of American and English surgeons, though until recently it undoubtedly has been such. S. W. W. Keen,[1] in a paper on the treatment of cancer of the rectum, alludes to this change of view and practice. He there states it as a general rule that all portions of the rectum and colon are amenable to the radical treatment, if other conditions than those of locality are not such as to forbid it. When the growth is in the lower part it may be reached by the perineal or sacral route; when too high for that, by laparotomy, or by both routes. If palliative treatment only is available, this writer recommends Maydl's inguinal colotomy. In that operation a good spur is formed by passing a glass rod through the mesentery close to the bowel, and resting upon the abdominal wall on each side of the wound. The bowel is then sutured to the edges of the wound. Lumbar colotomy is much less satisfactory. Before undertaking radical treatment the rectum should be thoroughly evacuated, a consummation by no means easy to achieve. The accumulation may be enormous above the growth. It may take from a week to a month to thoroughly empty the colon (Keen). The dangers of resections or amputations of the bowel are primary shock and hæmorrhage and infection of the wound. The colon is emptied to prevent the latter. Additional efforts may be made to disinfect the portion of bowel operated upon by the use of the drugs resorcin and salol, or salicylate of bismuth, by the employment of frequent and prolonged rectal irrigation, administered by the method Sir Thornley Stoker[2] advocates, by curetting of the foul rectal ulcer, which teems with organisms, or by a preliminary colotomy. Dr. Edward Taylor[3] urges the adoption of all the above methods except the last, which he does not adopt as a routine, but only if he fails otherwise to empty the colon. Keen recommends it in every case if the peritoneum has to be opened. Quénu[4] is even more emphatic, and regarding it as impossible by mere colotomy to asepticise the bowel, insists that both ends of the diseased organ should be carefully obliterated, in order that it may be removed *en masse*, like a cyst. If Maydl's colotomy were performed the lower segment of bowel could be cleansed by the injection of antiseptic fluids passed into the bowel through a catheter from above.

The method of removing the diseased bowel should be determined by the position and extent of the growth. Quénu[5] groups the cases into four classes: (1) Anal cancers; (2) cancers which are in the supra-sphincteric zone, above the levatores ani and infra-peritoneal; (3) those placed high up, from the level of the peritoneum below upwards to reach or involve the omega loop (of Treves; (4) extensive cancers reaching from the anus below a considerable distance upwards. For anal cancer the ordinary perineal excision of the rectum should be the method adopted. The glands of the groin are liable to be infected in this form, and should be removed if enlarged (Taylor[3]).

For the next class of cases, the supra-sphincteric, the perineal operation is giving place in modern practice to sacral operations. Taylor even goes so far as to state that "perineal excision of the rectum, as it has been performed, and as it is still described, is a bloody and barbarous procedure." He lays stress upon the careful way in which the attachments of the levatores ani to the bowel are to be separated, and the bowel cut across above, and later drawn down between, those muscles, and fixed to the anal mucous membrane, and also, as advocated by Heuston, fixed higher up by fixation sutures, to avoid tension, and passed between the bowel and the levatores. The posterior wound, which extends from the anus to the coccyx, is to be closed by sutures also passing through the levatores.

The results of operations upon the rectum for carcinoma have to be considered from the point of view of the completeness of extirpation they permit, and that of the perfection of functional repair they secure, and the perineal method has many faults from both these points of view. There is deficiency of room, the hæmorrhage is often free and difficult to arrest, and sphincteric action later is usually very imperfect Taylor). The sacral operation exposes the diseased parts much more efficiently, which is obviously a great advantage, allowing the healthy and diseased parts to be more easily differentiated, and the hæmorrhage to be more easily arrested. The method of sacral excision has been gradually evolved through the labours of Kocher, Kraske, and their followers. Thus, Kocher in 1874 made the first advance by removing the coccyx, and in 1885 Kraske removed not only the coccyx, but also a portion of the sacrum on the left side, from the third sacral foramen downwards to the left lower border. More extensive resections of the sacrum have been made by Kraske's followers, and by this route the uterus and ovaries and vaginal walls have been removed. In the more extensive resections the fourth sacral nerves are destroyed, and the anterior divisions of the third may be. As Taylor points out, this would be a great misfortune, for the sphincteric action of the levatores ani and the external sphincter and the sphincter of the bladder would be destroyed. Heinecke first introduced a method of temporary sacral resection, which others have modified; and there is much to be said in favour of these methods. Thirdly, the modification of approaching the rectum through an incision by the side of the sacrum has advocates, the sacrum itself not being divided.

Keen, in describing the technique after removing a portion of the sacrum, which he does by transverse division, if possible, below the fourth foramina, lays stress upon closing the peritoneum when opened before the bowel is divided above the cancer. He does this by suturing the border of the peritoneum to the anterior surface of the bowel, thus avoiding its infection. Then he removes glands, if affected. Then he amputates or resets the bowel. By amputation he means removal of the bowel down to the anus. In most cases of amputation he brings down the upper end of the bowel to the lower end of the resected spine, and there fixes it. After resection, the ends of healthy bowel may be sutured either with or without the aid of a bobbin; or Murphy's button may be used.

We have said that operations conducted on these lines are gaining popularity with English surgeons. In evidence of this the recent paper of Swinford Edwards[6] may be taken, and that of Littlewood.[7]

The mortality of the sacral operation is naturally greater than that of the perineal, and has been estimated as 20-25 per cent. Edwards writes: "I think that all will be agreed that in all cases where we feel morally certain that the entire disease can be removed, even at some considerable risk to life, the patient should be submitted to the radical operation." He gives a table of his cases, which comprises 14 cases, in six of which the coccyx and part of the sacrum were removed, in eight the coccyx only. There were two deaths—that is, a mortality of 14 per cent., two recurrences, 10 living and well, though in only three has more than two years elapsed since operation. Mr. Frederick Eve[8] reports a successful case of Kraske's operation, in which temporary resection of the sacrum and coccyx was performed by a median vertical incision to the level of the third sacral foramina and horizontal division at that level. The rectal stump was fixed to the skin at the upper part of the wound. A preliminary colotomy had been performed. Mr. Eve writes: "I would in future perform one or other of the modifications of the Kraske's operation, instead of the old operation, except for cases of disease limited to the lowest portion, or for circumscribed recent growths quite at the lower part of the middle portion of the rectum.

Mr. Littlewood's[7] paper contains a record of eight cases of rectal excision, three of them by modifications of Kraske's operation. All the cases were successful, and in one the ideal result of perfect union of the two portions of bowel across the gap left by excision, without a fistula, was obtained. The writer recommends the patient for operation to be placed prone on the table, with a block under pelvis and the thighs hanging below the end of the table and perpendicularly.

Mr. Jackson Clarke[9] published a successful case of Kraske's operation, in which the patient was well twelve months subsequently.

[1] Treatment of Cancer of the Rectum, the Therapeutic Gazette, Detroit, April 15, 1897. [2] British Med. Journ., Jan., 1895. [3] Treatment of Cancer of Rectum, Annals of Surgery, Ap., 1897. [4] The Operative Treatment of Rectal Cancer, Medical Week, March 5, 1897. [5] Presse Medicale, Nov., 1895. [6] The Removal of Cancer of the Rectum by Kraske's Method, B. M. J., May 15, 1897. [7] Lancet, Sept. 12, 1896. [8] Lancet, March 13th, 1897. [9] Lancet, Jan. 16, 1897.

### RHINOLOGY.

**Atrophic Rhinitis** is as fruitful a source of discussion as ever. At a discussion at the recent meeting of the American Laryngological Association in May, W. L. Casselberry[1] pointed out that there are fœtid and non-fœtid cases; and all stages of atrophy are observed. He recalled the fact that recently Belfanti and Della Vedova had found in the mucosa bacilli resembling those of diphtheria; Fränkel and Löwenberg, a coccus; Abel, a bacillus named by him bacillus mucosus; Wyatt Wingrave, various hyaloid bodies.

Dr. Jonathan Wright called attention to the fact that the disease was far more common in women than in men, and that its development seemed coincident with the menopause. After the menopause the condition generally improved. It had been suggested that the bacteria found in the nose in this affection might produce some ptomain, which by continued action on the nasal mucosa produced eventually the condition with which we are all so familiar.

Dr. S. O. Vander Poel, of New York, alluded to the serum therapy of Belfanti and Della Vedova. He had seen one subject of atrophic ozœna who contracted laryngeal diphtheria. Anti-toxin was injected at three different times, and the patient recovered. It was a remarkable fact that thereafter the ozœna was also greatly improved, the amount of discharge having been most markedly lessened.

Attilio De Simoni[2] draws attention to the unvarying presence of the bacilli of diphtheria in ozœna. The observations generally accepted on the parasitic nature of ozœna have recently been modified by the researches of Belfanti and Della Vedova, communicated to the Royal Academy of Turin in March last. The exact nature of the large pneumococcus of Löwenberg—although the latter, Marano, Abel, Paulsen, and others regard it as the specific element of ozœna in contradistinction to the latest researches of Belfanti and Della Vedova, who found and described a bacillus bearing all resemblance to the microbe of diphtheria, and only differing from it in degree of virulency—still remains to be established. The latter base their conclusions not alone on the constant concurrence of these bacilli in ozœna secretions, but on the clinical experience of 36 cases, either cured or improved by anti-diphtheritic serum treatment. According to the author, this fact in itself goes far to confirm the specific nature of the bacilli of Belfanti, &c. To further substantiate this he examined 25 cases of ozœna according to the methods described by Belfanti. In all of these, the specimens being stained with Gram-Weigert solution, on repeated examinations he invariably found a small, slender bacillus, almost fusiform in shape, one extremity larger than the other, at times straight, and again bent at a right angle, isolated or in groups, with a tendency to a parallel arrangement. He further lays great stress on the staining methods employed. Whilst those coloured with Gram-Weigart solution demonstrated the bacilli in abundance, the latter were wanting, or but sparse in quantity, in others stained with the usual methods of colouring adopted in bacteriological research. At the same time, the author devoted attention to Löwenberg's diplococcus, using the simple method of Ziehl. Thus, in all cases, he was able to find the diplococcus present, most numerous in the secretion of those cases of ozœna that had been neglected, or where measures of cleanliness had not been applied. Repeated examination of small pieces of the mucous membrane, removed by scraping, and treated according to Belfanti's method, always revealed the presence of the diphtheritic bacilli, and never that of the diplococcus. According to numerous observations, the latter have only been found either free in the secretion, or but adhering to the mucous membrane. According to this fact, and the presence of both types of micro-organisms, no deductions can as yet be formulated. Time and clinical experience alone can decide what influence they have in the etiology of ozœna.

Some points of importance on the treatment of ozœna are mentioned by Ralph Seiss.[3] Among drugs which have given good results as stimulants in the treatment of this disease, thymol, in his opinion, still continues in the first rank; it may be used in watery solution with alcohol and glycerin, but is much better employed dissolved in one of the petroleum oils; albolene, glymol, or benzoinol may be used, the thymol being in the proportion of from three to ten grains per ounce. The former strength can be borne by most patients; the latter only by a few in whom tolerance has been established by much previous treatment. Frontal headache, lacrymation, or a severe stinging sensation in the nose should always be regarded as indications for the use of a weaker solution. Numerous drugs may usefully be employed in combination with thymol; among them menthol, pine-needle oil, and eucalyptus, in the strength of from 1 to 3 per cent. Sanguinaria and galangal were once much used as stimulants in this disease, but they are exceedingly apt to act as severe irritants, and owing to this and their unpleasant taste have been largely abandoned. Next to thymol europhen has given the most satisfaction. Cinnamon oil has been shown to be the most decidedly antiseptic of the volatile oils, and is particularly valuable in conditions of nasal and pharyngeal sepsis; it may be used in the form of a spray, from 3 to 10 per cent. being added to the ounce of albolene. It appears to be entirely non-irritating, produces a grateful feeling in the nose, and its odour is pleasant and not persistent.

Seiss observes that stimulation may be as easily overdone in cirrhotic catarrh by drugs as by mechanical means. MacDonald states that "it is somewhat doubtful whether we are any more justified in stimulating the nasal mucous membrane in atrophic rhinitis than a cirrhotic liver." It cannot too often be reiterated that all violent measures in this disease defeat themselves by causing the very pathological processes which they are intended to cure. Seiss has seen several cases in which extensive intranasal operations had appeared to light up the atrophic process, and all operations on the turbinated tissues are to be regarded as at most unfortunate necessities; and Jonathan Wright has had a similar experience, and forcibly points out how, in vascular engorgement, cauterisation or other intranasal operation does not restore the efficiency of the damaged muscular apparatus, but by the formation of a scar favours the tendency to fibrosis.

[1] Med. Rec., May 22, 1897. [2] Boll. delle Malatt. dell Orecchio, &c., Aug., 1896; Jour. of Laryngology, March, 1897. [3] Med. News, Nov. 28, 1896; Therap. Gazette, April 15, 1897.

